# Transcriptome analysis of the venom gland of the scorpion *Scorpiops jendeki*: implication for the evolution of the scorpion venom arsenal

**DOI:** 10.1186/1471-2164-10-290

**Published:** 2009-07-01

**Authors:** Yibao Ma, Ruiming Zhao, Yawen He, Songryong Li, Jun Liu, Yingliang Wu, Zhijian Cao, Wenxin Li

**Affiliations:** 1 State Key Laboratory of Virology, College of Life Sciences, Wuhan University, Wuhan, 430072, PR China

## Abstract

**Background:**

The family Euscorpiidae, which covers Europe, Asia, Africa, and America, is one of the most widely distributed scorpion groups. However, no studies have been conducted on the venom of a Euscorpiidae species yet. In this work, we performed a transcriptomic approach for characterizing the venom components from a Euscorpiidae scorpion, *Scorpiops jendeki*.

**Results:**

There are ten known types of venom peptides and proteins obtained from *Scorpiops jendeki*. Great diversity is observed in primary sequences of most highly expressed types. The most highly expressed types are cytolytic peptides and serine proteases. Neurotoxins specific for sodium channels, which are major groups of venom components from Buthidae scorpions, are not detected in this study. In addition to those known types of venom peptides and proteins, we also obtain nine atypical types of venom molecules which haven't been observed in any other scorpion species studied to date.

**Conclusion:**

This work provides the first set of cDNAs from *Scorpiops jendeki*, and one of the few transcriptomic analyses from a scorpion. This allows the characterization of a large number of venom molecules, belonging to either known or atypical types of scorpion venom peptides and proteins. Besides, our work could provide some clues to the evolution of the scorpion venom arsenal by comparison with venom data from other scorpion lineages.

## Background

Based on cladistic morphological analysis, the extant scorpions can be phylogenetically divided into 14 families[1]. All scorpions possess a homologous venom apparatus which consists of the vesicle holding a pair of venom glands and the hypodermic aculeus used to inject the venom[2]. Scorpion venom is a combinatorial library of peptides and proteins which could cause toxicological responses and can be candidates for drug design and development[3]. The general compositions of scorpion venoms vary among different families. For instance, in a comparative LC/MS analysis of two scorpion species from the families Buthidae and Ischnuridae, vast abundance difference was observed in venom components with molecular size from 5000 to 10,000 Da[4]. Furthermore, such differences in venom compositions could also be observed from genus to genus, and even between different species within a genus[5,6].

Hundreds of venom peptides and proteins have been characterized from various scorpion species[7]. It is noteworthy that most of these venom molecules are obtained by either bioassay-guided fractionation or PCR-based methods conducted with cDNA libraries. Due to their medical importance, most research performed to date has focused on Buthidae scorpions. Buthid venoms mainly consist of four different families of neurotoxins which specifically target ion channels, including sodium channels, potassium channels, chloride channels, and calcium channels [8-10]. However, in contrary to buthids, little attention has been paid to the other thirteen non-Buthidae families. As several classes of venom peptides and proteins from non-Buthidae scorpions were shown to possess unique primary sequences and biological activity, it is worth exploring the venom compositions of non-Buthidae scorpions[4].

The scorpion *Scorpiops jendeki *is distributed in Yunnan province, Southwest China[11]. It was once considered to be a member of the family Scorpiopidae, but now it is classified into the family Euscorpiidae after a very thorough phylogenetic analysis[1]. The Euscorpiidae family is among the most widely distributed groups of extant scorpions, and it covers Europe, Asia, Africa, and America[1]. Euscorpiids are considered to be harmless scorpions which possess no threat to human health. So far, euscorpiid venoms haven't been studied yet.

Different from bioassay-guided isolation, an "-ome" approach such as transcriptomic or proteomic analysis could help uncover the real diversity of scorpion venom components. Not only known types of venom peptides and proteins but also atypical venom molecules could be obtained by such an approach. Until now, proteomic studies have been employed in assessing the diversity of venom compositions from several scorpion species[12]. Only one transcriptomic analysis has been conducted on the venom gland of a scorpion[13]. An extensive knowledge of venom compositions from different scorpion species is helpful in understanding the envenomation and providing candidate molecules for drug development. Furthermore, comparative analysis of venom constituents from different scorpion lineages could also provide a clue to the evolutionary track of scorpion venom arsenal, as illustrated in the snake venom systems [14-16].

In this work, we carried an EST approach to overview the transcriptome of the *Scorpiops jendeki *venom gland. A great number of venom peptides and proteins, belonging to known and atypical toxin types, were identified through the first transcriptome study on the venom gland of a Euscorpiidae scorpion. Besides, venom data comparison among different scorpion lineages provides some clues to the evolutionary track of the scorpion venom arsenal.

## Results

### EST sequencing and clustering

**Figure 1:**
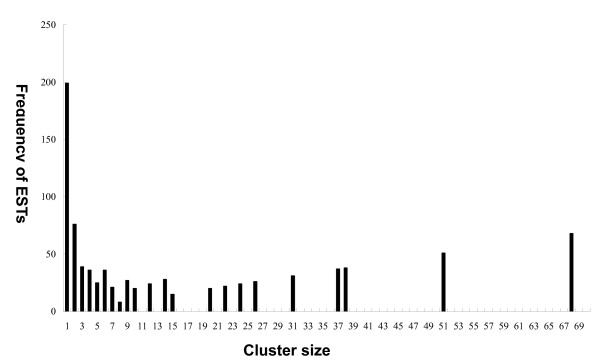
**ESTs distribution by cluster size**. For instance, there are 6 clusters of size 6, accounting for a sum of 36 ESTs.

The titer of the non-amplified cDNA library is 3.5 × 10^6 ^cfu/ml with more than 98% recombination efficiency. The random sequencing of this library gave readable sequences for a total of 871 clones. After being processed as described in the "Materials and Methods" section, the high quality EST sequences were submitted into the dbEST (accession numbers: GH547439–GH548309). The average length of these processed sequences was 546 bp. After being grouped with stringent parameters, the ESTs formed 293 clusters of unique sequences, including 199 singletons and 94 contigs consisting of two or more ESTs (Figure 1). In this study, the terms "contig" and "singleton" were used as the same meaning as described in Egassembler[17].

To attempt a functional classification of these unique sequences, we compared the consensual cluster sequences against SWISS-PROT and GenBank NCBI databases by BLAST algorithms. 208 clusters (644 ESTs) provided significant hits (Expect value < e-4), whereas the other 85 clusters (227 ESTs) hadn't good matches (Table 1). Among the matched clones, 59 clusters (445 ESTs) are deduced to be secretory peptides and proteins. For the non-match set, the longest ORFs from each cluster were predicted and screened for possible signal peptides. Among the non-matched clones, 39 clusters (175 ESTs) are supposed to possess a signal peptide, and 26 clusters (27 ESTs) haven't ORF found. Since the cDNA library was not amplified, the clone number was expected to reflect the actual prevalence of a given transcript in the original biological sample. So transcripts related to secretory proteins, including venom peptides and other physiological proteins, make up more than 70% of total ESTs of the *Scorpiops jendeki *venom gland.

**Table 1:** Distribution of 293 clusers assembled from the scorpion *Scorpiops jendeki*

**Category**	**Secretory (clusters/ESTs)**	**Non-Secretory** **(clusters/ESTs)**	**Non ORF** **(clusters/ESTs)**
Matching sequences			
Similar to venom peptide transcripts	33 (359)		
Not similar to venom peptide transcripts	26 (86)	149 (199)	
Non-matching sequences	39 (175)	20 (25)	26 (27)
			
Total	98 (620)	169 (224)	26 (27)

### Known toxin types

10 known toxin types have been characterized from the scorpion *Scorpiops jendeki*. They are encoded by 359 ESTs (33 clusters), accounting for approximately 40% of the total venom gland transcripts (Table 1).

#### α-KTx

**Figure 2:**
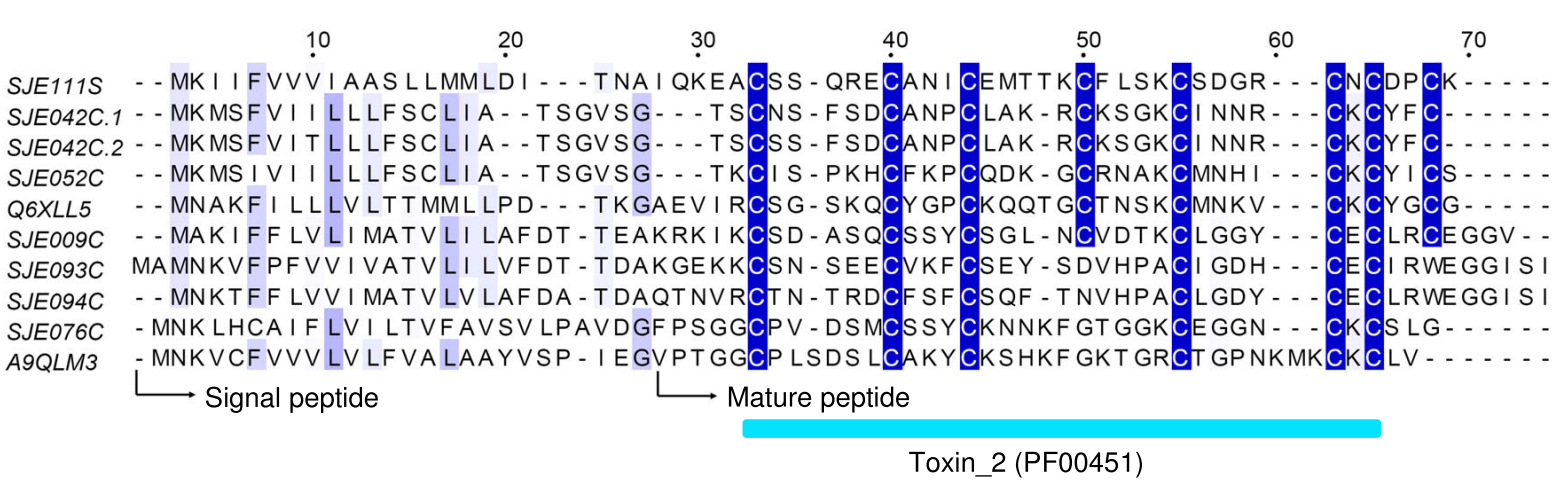
**Sequence alignment of α-KTxs**. SJEs are clusters from this work. The others are Q6XLL5 (alpha-KTx 6.10, *Opistophthalmus carinatus*), and A9QLM3 (LmKTx8, *Lychas mucronatus*).

α-KTxs have a wide phylogenetic distribution, and have been obtained from almost all scorpion species studied so far[8]. The newly identified α-KTxs are encoded by seven clusters (six contigs and one singleton, 45 ESTs). Among them, three clusters (SJE076C, SJE093C and SJE094C) code for α-KTxs contrained by 3 disulfide bridges, whereas the other clusters for α-KTxs with four disulfide bridges (Figure 2). For some toxins, the fourth disulfide bridge is of great importance in reaching the correct bioactive conformation[18]. They share the Toxin_2 domain (Pfam: PF00451) with other previously characterized scorpion short-chain toxins which could act on *shaker*-related channels or Ca^2+^-activated K^+^-channels[8].

It is noteworthy that SJE042C consists of two almost identical ESTs differing by only a few nucleotides. The translated sequences are named SJE042C.1 and SJE042C.2, respectively. Similar phenomenon has also been extensively observed in other types of venom peptides and proteins discussed followingly. The possibility that these minor differences are derived in the course of cDNA library construction and sequencing could be excluded, as the phenomenon can hardly be observed in the clusters encoding common cellular proteins[19]. Such subtle differences in EST sequences reflect the polymorphism of scorpion venom peptide genes[20].

Interestingly, although SJE009C have four disulfide bridges, it shows closer relationship with SJE093C and SJE094C, the α-KTxs with three disulfide bridges. This highlights the evolutionary relationship between α-KTxs with 3 disulfide bridges and those with 4 disulfide bridges.

#### Scorpine-like peptide

**Figure 3:**
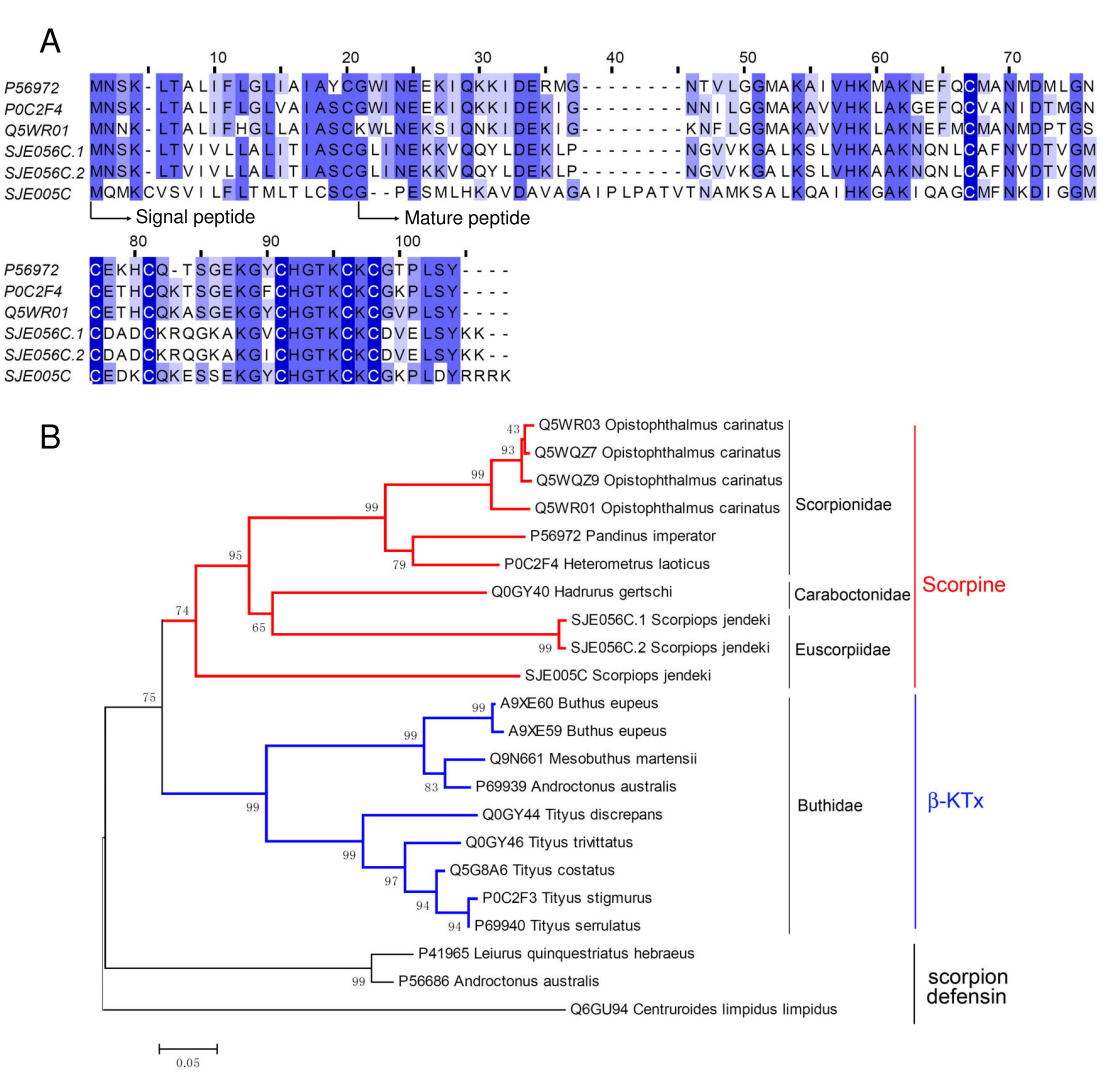
**Scorpines**. (A) Sequence alignment of scorpines. SJEs are clusters from this work. The others are P56972 (Scorpine, *Pandinus imperator*), P0C2F4 (Heteroscorpine-1, *Heterometrus laoticus*), and Q5WR01 (Opiscorpine-2, *Opistophthalmus carinatus*). (B) Phylogeny analysis of β-KTxs and scorpines from scorpion venoms. To minimize confusions, all proteins from previous work are represented by their SWISS-PROT accession numbers. Scorpion defensins are used to root the phylogeny tree.

Due to poor knowledge of their functions, scorpine-like peptides were once classified into "orphan" venom components[21]. Several recent studies have demonstrated that scorpines possess anti-malaria and antimicrobial activities[22,23]. Besides, they can also function as K^+ ^channel blockers[24]. Two clusters of scorpine-like peptides, SJE005C and SJE056C, were identified in this work (Figure 3A). They show great similarity with scorpine-like peptides obtained from other scorpion species, such as *Heterometrus laoticus *and *Hadrurus gertschi*[13,25].

Scorpine-like peptides show obvious sequence similarity to β-family of KTxs. But distinct to β-KTxs, they don't possess a putative short pro-sequence following the signal peptide[21]. Until now, all scorpine-like peptides are exclusively obtained from non-Buthidae scorpions, whereas all β-KTxs are from Buthidae scorpions (Figure 3B). The Scorpine-like peptide Tco 41.46-2, which is isolated from *Tityus costatus *(Buthidae), should be classified into β-KTxs, based on sequence similarity and the presence of a pro-peptide[24]. As scorpion neurotoxins are paralogous genes of defensins, scorpion defensins were used to root the phylogeny tree[26,27]. The reconstructed phylogeny relationship strongly suggests that β-KTxs and scorpine-like peptides share a common ancestor before the lineage split between Buthidae and the non-Buthidae families. After the lineage split, β-KTxs and scorpine-like peptides evolve independently in different scorpion families.

#### Calcine

**Figure 4:**
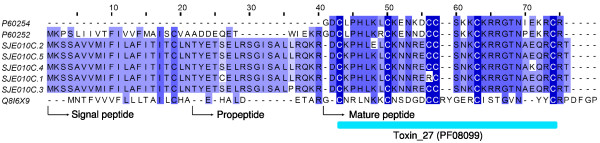
**Sequence alignment of calcines**. SJEs are clusters from this work. The others are P60252 (Opicalcin-1, *Opistophthalmus carinatu*), P60254 (Maurocalcin, *Scorpio maurus palmatus*), and Q8I6X9 (BmCa-1, *Mesobuthus martensii*).

Calcines can act against ryanodine receptors, a type of intracellular endoplasmic/SR (sarcoplasmic reticulum) calcium release channels distributed in cardiac and skeletal muscle[28,29]. They penetrate into the cell via interaction with membrane lipids[30]. Structurally, they are characterized to harbor an inhibitor cystine knot fold, which is shared by a large number of polypeptides from diverse animal species[31,32]. Based on their cell-penetrating ability, calcines have been used as a non-toxic drug carrier to overcomes drug resistance in cancer therapy[33]. In this study, one cluster (SJE010C, 51 ESTs) were identified to encode calcines (Figure 4). There are five variants (SJE010C.1-SJE010C.5) with subtle differences. Interestingly, the cysteine pattern has been changed in SJE010C.1. These newly identified calcines also harbor the Toxin_27 domain (Pfam: PF08099).

#### Cytolytic peptide

The first cytolytic linear peptide, named IsCT, was got from the scorpion *Opisthacanthus madagascariensis*, a member of the family Scorpionidae[34,35]. Then this type of venom peptides were later found in the scorpion *Mesobuthus martensii *(Buthidae)[36]. Their precursors consist of a signal peptide, a mature peptide and a C-terminal propeptide rich in acidic amino acids. Cytolytic peptides possess broad activity spectra against microbes and hemolytic activity. They are suggested to lyse cell membranes via pore formation or destabilization of membrane phospholipid packing, based on their amphiphilic α-helical structures[37].

**Figure 5:**
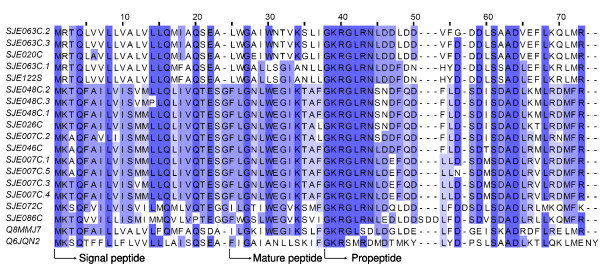
**Sequence alignment of cytolytic peptides**. SJEs are clusters from this work. Q8MMJ7 is cytotoxic linear peptide IsCT from the scorpion *Opisthacanthus madagascariensis*, and Q6JQN2 is BmKn2 from *Mesobuthus martensii*.

In the transcriptome of the *Scorpiops jendeki *venom gland, cytolytic peptide precursors are the most highly expressed venom peptide transcripts. There are nine clusters (eight contigs and one singleton, 88 ESTs), representing approximately 10% of venom gland mRNAs. In contrast, in our previous investigation of *Mesobuthus martensii *venom, cytolytic peptides were observed at a rather low expression level (data not shown). Based on sequence similarity, the cytolytic peptides obtained in this study are divided into two clades: SJE020C, SJE063C and SJE122S form one clade; while the other clade consists of SJE007C, SJE026C, SJE046C, SJE048C, SJE072C and SJE086C(Figure 5). Translated sequences from each clade are almost identical in the signal peptide region, but rather variable in mature peptide and propeptide regions.

#### Trypsin inhibitor like (TIL) peptide

**Figure 6:**
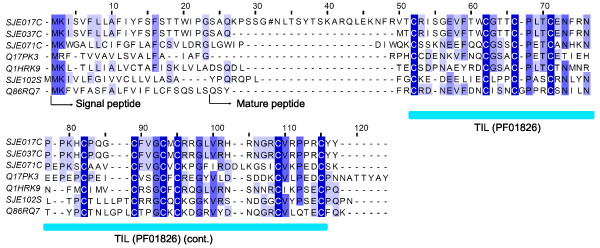
**Sequence alignment of trypsin inhibitor like (TIL) peptides**. SJEs are clusters from this work. The others are Q17PK3 (Cysteine-rich venom protein, *Aedes aegypti*), Q1HRK9 (TIL domain-containing cysteine-rich salivary secreted peptide, *Aedes aegypti*), and Q86RQ7 (Venom peptide BmKAPi, *Mesobuthus martensii*).

A trypsin inhibitor like venom peptide, BmKAPi, has previously been characterized from the scorpion *Mesobuthus martensii*[38]. The trypsin inhibitor like domain (Pfam: PF01826) contains ten cysteine residues that form five disulphide bonds[39]. However, the exact function of trypsin inhibitor like peptides from scorpion venoms hasn't been clarified[40]. Four clusters (three contigs and one singleton, 15 ESTs) were identified to encode trypsin inhibitor like peptides (Figure 6).

Of note, SJE017C is almost identical to SJE037C, except for a 72 bp insertion into the former. Which molecular mechanism causes this phenomenon would depend on uncovering their genomic organizations and structures. Interestingly, a nonsense mutation in the 72 bp insertion of SJE017C results in a premature stop codon. Three ESTs in SJE017C represent different transcripts of the same gene, as they are not completely identical. So the possibility of an error in the sequencing is excluded. Resequencing these three clones further supports the nonsense mutation. So the cluster SJE017C may represent a pseudogene.

Secretory peptides with trypsin inhibitor like domain can also be found in the venom glands of mosquito[41-43]. They function as serine protease inhibitors or antimicrobial peptides[44,45]. So convergent evolution has repeatedly selected genes coding for proteins containing the trypsin inhibitor like cysteine rich domain as templates for venom molecules[46].

#### Lysozyme

**Figure 7:**
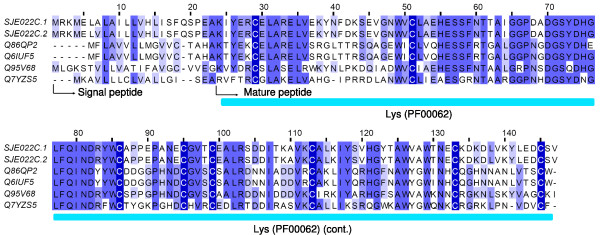
**Sequence alignment of lysozymes**. SJEs are clusters from this work. The others are Q86QP2 (Lysozyme, *Branchiostoma belcheri tsingtauense*), Q6IUF5 (Lysozyme C, *Branchiostoma belcheri tsingtauense*), Q95V68 (Lysozyme, *Ornithodoros moubata*), and Q7YZS5 (Lysozyme, *Triatoma infestans*).

The known lysozymes within the animal phyla are classified into 3 different types: chicken type (c-type), invertebrate type (i-type), goose-type (g-type)[47]. A c-type lysozyme has previously been partially sequenced in a proteomic analysis of the venom from the scorpion *Tityus stigmurus*[5]. In this work, one cluster (SJE022C, 9 ESTs) was identified to code c-type lysozymes (Figure 7). They are greatly homologous to c-type lysozymes from other sources. Generally, lysozymes play an important defense role in the innate immunity. The exact biological role of lysozymes from scorpion venoms remains to be explored, as they have a relatively high expression level. As demonstrated in a previous report, lysozyme can also function as the termite egg recognition pheromone[48].

#### La1-like peptides

**Figure 8:**
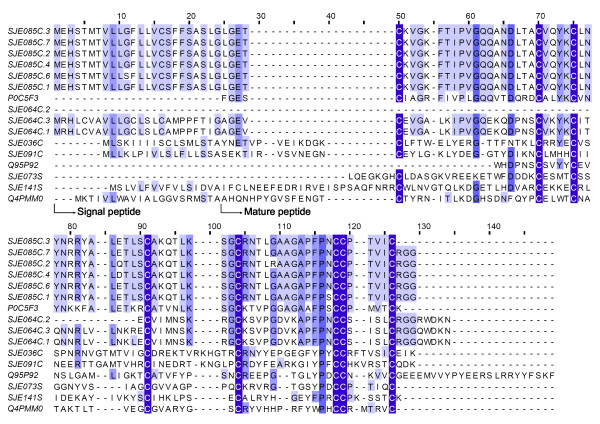
**Sequence alignment of La1 like peptides**. SJEs are clusters from this work. The others are P0C5F3 (Venom peptide La1, *Liocheles australasiae*), Q4PMM0 (Putative secreted salivary protein, *Ixodes scapularis*), and Q95P92(TXLP1, *Mesobuthus martensii*).

La1 is the most abundant venom peptide obtained from the scorpion *Liocheles australasiae*[4], which was once considered to be a member of the family Hemiscorpiidae, but now has been classified into the family Ischnuridae[1]. Acturally, this type of venom peptides was firstly characterized from the scorpion *Mesobuthus martensii *at the transcript level. Until now, there have been no clues to their biological function. This work revealed six clusters of La1-like peptides, including four contigs and two singletons (Figure 8). In terms of primary sequence similarity and the position of eight cysteines, they are homologous to several known peptides, including secretory peptides from the salivary gland of *Ixodes scapularis *ticks[49]. This demonstrates that La1-like peptides have an ancient origin[50].

#### Opistoporin like peptide

**Figure 9:**
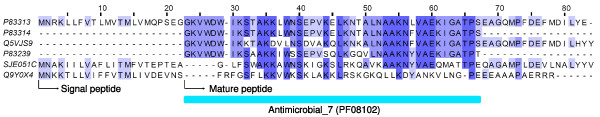
**Sequence alignment of Opistoporin like peptides**. SJEs are clusters from this work. The others are P83313 (Opistoporin-1, *Opistophthalmus carinatus*), Q5VJS9 (Opistoporin4, *Opistophthalmus carinatus*), Q9Y0X4 (Bradykinin-potentiating peptide BmK3, *Mesobuthus martensii*), P83314 (Opistoporin-2, *Opistophthalmus carinatus*), and P83239 (Pandinin-1, *Pandinus imperator*).

The cluster SJE051C is identified to encode an antimicrobial peptide which shares the Antimicrobial_7 domain (Pfam: PF08102) with opistoporins and pandinin (Figure 9). Opistoporins are antimicrobial peptides isolated from the venom of the South-African scorpion *Opistophtalmus carinatus*, whereas pandinin is from the scorpion *Pandinus imperator*[51,52]. These peptides form essentially amphipathic helical structures and demonstrate high antimicrobial efficiency against Gram-negative and Gram-positive bacteria. Besides, it is also homologous to BmKbpp, which is a bradykinin-potentiating peptide obtained from the Chinese scorpion *Mesobuthus martensii*[53].

#### Anionic peptide

**Figure 10:**
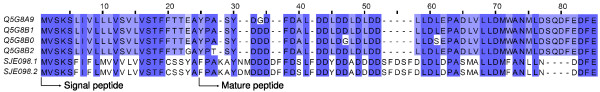
**Sequence alignment of anionic peptides**. SJEs are clusters from this work. Q5G8B2, Q5G8A9, Q5G8B1, and Q5G8B0 are different anionic peptides from the scorpion *Tityus costatus*.

Anionic peptides have previously been characterized from *Mesobuthus martensii *and *Tityus costatus*, two scorpion species from the family Buthidae[36,54]. As the name suggests, this type of venom peptides are rich in acidic amino acid residues (aspartic acid and glutamic acid). A cluster (SJE089C, 2 ESTs) was identified to encode anionic peptides (Figure 10). It is not clear what their biological role is. As the vast majority of scorpion venom peptides are basic, anionic peptides are suggested to play a part in balancing the pH value of scorpion venom liquid[36].

#### SPSVs (serine proteases from scorpion venoms)

**Figure 11:**
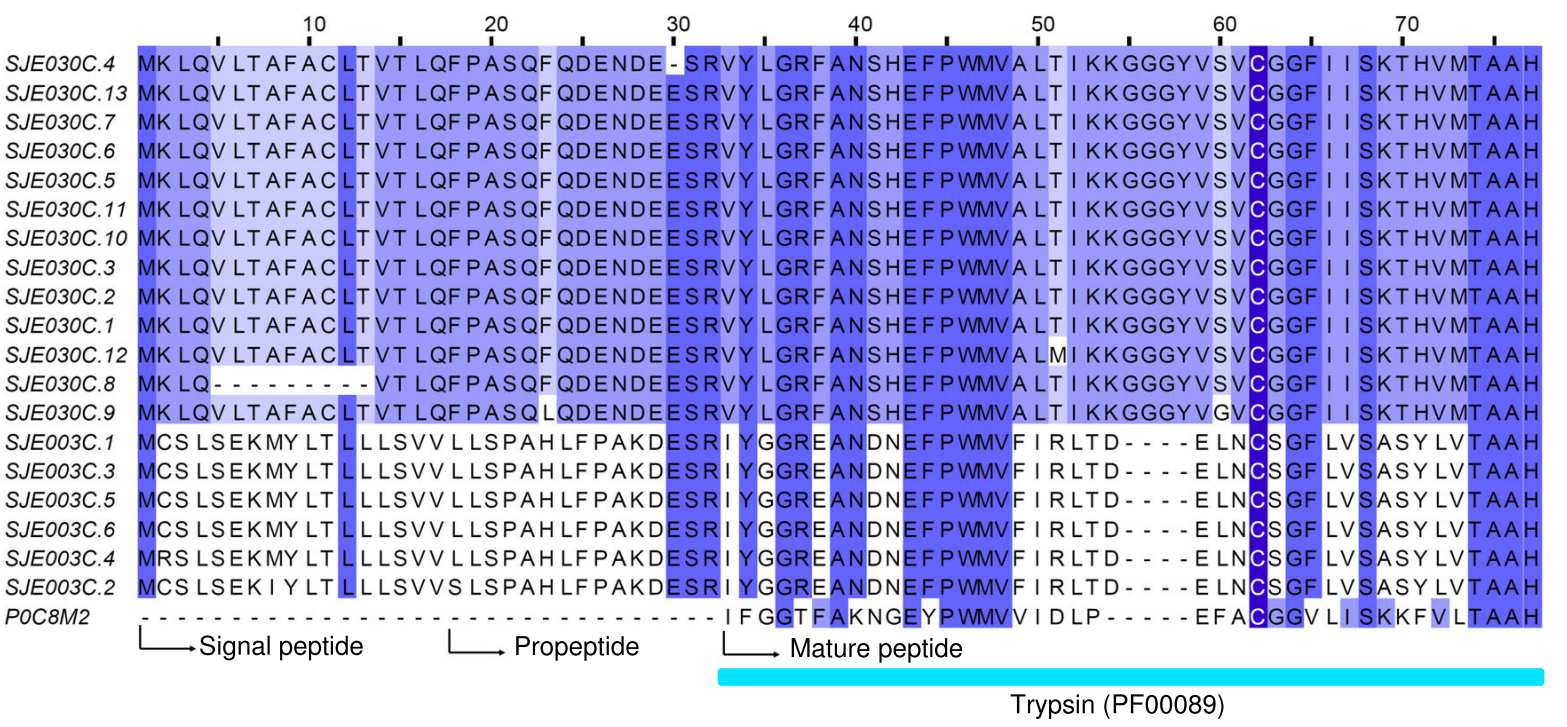
**N-terminal sequence alignment of SPSVs (serine proteases from scorpion venoms)**. SJEs are clusters from this work. P0C8M2 is BMK-CBP obtained from the scorpion *Mesobuthus martensii*.

To date, most studies performed on scorpion venoms have focused on isolation and characterization of neurotoxins and antimicrobial peptides. Although proteolytic enzyme activities have been detected in the venom of several scorpion species for a long time[55,56], the first serine proteinase-like protein has recently been purified and partially sequenced in a screen for drug candidates targeting cancer cells[57]. Two clusters (SJE003C and SJE030C, 78 ESTs) were identified to encode serine proteases from scorpion venoms, here named SPSVs (Figure 11). As their precursors are composed of more than 200 amino acid residues, they represent important parts of the venom proteins with high molecular weight (>20 KDa). SPSVs may be involved in post-translational processing of other venom peptides, and can also function as "spreading factors" in order to facilitate the spread of other venom peptides[56].

### The atypical possible toxin types

In addition to those known types of venom peptides and proteins as described above, there are also several clusters supposed to encode novel venom peptide types, base on their high expression level and the presence of the signal peptide.

**Figure 12:**
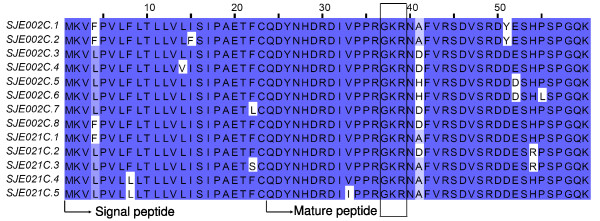
**Sequence alignment of jendins**. SJEs are clusters from this work. The boxed "GKR" part of the translated sequences represents typical processing signal.

A highly expressed type of venom peptides was identified to be encoded by clusters SJE002C and SJE021C containing 37 and 22 ESTs each (Figure 12). Here we named them jendins. They have no hit found against any public database, indicating that jendins are an atypical peptide types from scorpion venoms. Jendin precursors consist of a signal sequence of 23 residues and a premature peptide of 37 residues. The premature peptide has a typical processing signal (Gly-Lys-Arg) at positions 14–16[36]. It remains to be explored whether jendins have a similar post-translational processing as cytolytic peptides[34,58]. Furthermore, their biological function remains to be investigated.

Besides, there are several medium-abundant clusters which are deduced to encode eight novel types of scorpion venom peptides [see Additional file 1]. They are either cysteine-free or cysteine-rich. Similar to jendins, they have not homologs found from public database. The presence of atypical venom peptides and proteins indicates that scorpion venoms are a rather complex pool, and multiple currently unkown types of venom peptides and proteins remain to be characterized from different scorpion lineages.

### Common cellular protein ESTs

The scorpion venom gland is a specialized organ for synthesizing and secreting venom components. As demonstrated in *Scorpiops jendeki*, transcripts for different types of venom peptides and proteins account for more than 50% of the full transcriptome. So it is interesting to overview the physiological state of the venom gland when it highly expresses venom peptides and proteins.

**Figure 13:**
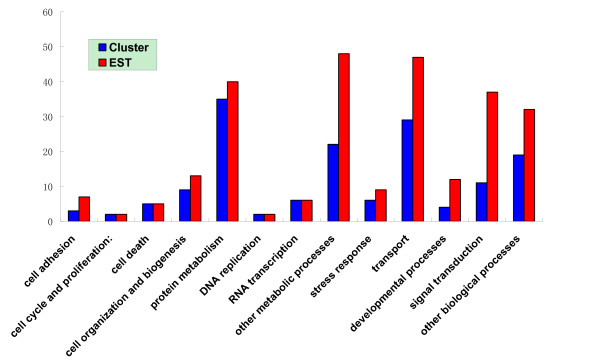
**Functional characterization of ESTs and assembled clusters from the *Scorpiops jendeki *venom gland**. The vertical axis shows the number of ESTs or clusters.

Among the matched non-toxin transcripts, 153 clusters (260 ESTs) have their physiological function found (Figure 13). Most of these clusters consist of only one or a few ESTs. Although the limited sequencing data of this study is far from the complete description of *Scorpiops jendeki *venom gland, it could be used to roughly estimate the ralative redundance of each category. Genes, which are involved in RNA transcription and especially protein metabolism, are highly expressed in the *Scorpiops jendeki *venom gland. The molecules related to protein metabolism are mainly diverse kinds of ribosomal proteins responsible for protein synthesis. Besides, protein synthesis and other metabolic process are highly energy-consuming, and protein processing and transporting is also intense for the newly-synthesized venom peptides. Accordingly, high expression levels are also observed in the gene sets within the transport category which are mainly responsible for the energy generation and protein sorting.

## Discussion

During more than 400 million years of evolution, scorpions have developed an efficient venom arsenal, composed of extremely diverse active components, to prey captures and deter competitors. The venom molecules are able to induce both toxicological and immunological responses, and also offer a tremendous resource for use in drug development. Usually transcriptome or proteome approach is employed to explore the complexity of venom components. Several recent studies performed on many venomous species demonstrate that venom proteome and transcriptome depart in their relative abundances of different toxin families[59,60]. However, the ESTs-based transcriptome strategy has been shown to be effective in uncovering the real diversity of venom compositions[13,61]. Not only sequences of known toxin types but also atypical venom molecules could be characterized by such a transcriptomic approach.

In this work, we have employed a transcriptomic approach to investigate possible venom components from the scorpion *Scorpiops jendeki*. Before RNA extraction, the scorpion specimens are milked by electrical stimulation. So the gene expression profiling obtained in this work represents the activated-state transcription of the venom glands. The transcripts for possible venom constitutes make up approximately 50% of the *Scorpiops jendeki *transcirptome. It is much higher than that observed for the scorpion *Hadrurus gertschi *(approximately 30%)[13]. Such difference may be attributed to genetic variations[12]. This work could be used in comparative studies of gene expression profiling among different scorpion species.

Among different scorpion venoms, there are great variability in proportion of different types of venom peptides and proteins. A previous study conducted a comparative proteomic analysis of scorpion venom components with the method of mass finger print comparison among three different *Tityus *venoms[12]. It shows that the proportion of molecular weight distribution is rather variable among *Tityus cambridgei*, *Tityus costatus *and *Tityus discrepans*. Until now, there is only one transcriptome study of scorpion venom glands[13]. In the transcriptome of the *Hadrurus gertschi *venom gland, α-KTxs and scorpine-like peptides are most highly expressed, accounting 17.7% of the total ESTs. However, the most prevalent types of venom peptides and proteins are cytolytic peptides and SPSVs in *Scorpiops jendeki*. Approximately 19% of the total ESTs encode for the precursors of these two types of molecules. It is noteworthy that the four types (SPSVs, La1-like peptides, calcines, and jendins), with a high expression level in *Scorpiops jendeki*, were not detected in *Hadrurus gertschi *at all. Although different types of venom molecules couldn't arise in proteins at the same level of their mRNAs, we could definitely conclude that there is great difference in venom compositions between *Scorpiops jendeki *and *Hadrurus gertschi*. Furthermore, the venom compositions of *Scorpiops jendeki *must be different from that of Buthidae scorpions, whose major groups of venom constitutes are neurotoxins affecting Na^+ ^channels (NaScTxs) and K^+ ^channels (KTxs).

Great diversity has also been observed in primary sequences of most highly expressed venom peptides and proteins. We can exclude the possibility that such diversity is caused by the artifact in cDNA library construction or DNA sequencing. A negative control is that 31 ESTs from SJE009C encode one identical translated sequence. Such diversity may mainly be attributed to variations in scorpion population, as the cDNA library was constructed with the RNA extracted from about 50 specimens. However, a previous study demonstrates that such polymorphism could also arise at the level of individual scorpion[20]. Whatever, such diversity extensively observed in different types of venom peptides and proteins reflects the dynamic process of diversification. It is beneficial for the survival of scorpions, as the more and more complex venom arsenal could meet their demands for interaction with their prey, predators, and competitors[62].

The most striking observation of this study is the absence of NaScTxs in *Scorpiops jendeki*. This phenomenon has also been observed in the non-Buthidae scorpion *Hadrurus gertschi *(Caraboctonidae), on which a transcriptomic analysis has been conducted[13]. NaScTxs are peptides of 58–76 residues in length and characterized to possess a structure core, named Cysteine-Stabilized α/β motif (CS-αβ), tightly packed by three conserved disulfide bridges[9]. They are a major group of venom components from Buthidae scorpions. NaScTxs and KTxs are suggested to evolve from a common progenitor, based their similarities in gene organizations, intron features and structure cores[20]. But their evolutionary history is difficult to reconstruct, due to high diversity of each toxin types[63,64]. Similar to NaScTxs, KTxs are also defined by the presence of the conserved CS-αβ motif[8]. Distinct to NaScTxs, KTxs have been obtained from most scorpion species, both Buthidae and non-Buthidae, currently under investigated. The difference between the phylogeny distribution of NaScTxs and KTxs could provide some clues to their evolutionary relationship.

Until now, many types of venom peptides and proteins have been obtained from diverse scorpion species. Some types are found to be widely distributed among scorpion species from different families, in case of α-KTxs. However, some other types appear to be restricted to particular scorpion lineages. For instance, jendins haven't been detected in other scorpion species. Scorpine-like peptides have not been obtained from Buthidae scorpions, although some Buthidae scorpion species have been extensively studied. So far transcriptome studies are lacking even for the medically imprtant Buthidae scorpions. However, this work implies that the presence of additional, atypical toxin types in many scorpion lineages is most likely. The presence of these common and uncommon venom molecules among different lineages reflects the dynamic evolutionary process of the scorpion venom arsenal. In order to depict such a process, extensive studies should be conducted on diverse scorpion species, especially from the non-Buthidae families.

## Conclusion

In conclusion, we conducted a transcriptomic analysis of *Scorpiops jendeki *venom gland. *Scorpiops jendeki *belong to the family Euscorpiidae whose venoms have never been investigated. So our work greatly expanded the current knowledge of scorpion venoms. We obtained ten known types and nine atypical types of venom peptides and proteins. These molecules provide a rich hitherto unexplored resource for drugdevelopment. Besides, some clues can be provided into the evolution of scorpion venom arsenal by comparing the presence of common and umcomon types of venom peptides and proteins among different scorpion lineages.

## Methods

### cDNA library construction

50 specimens of *Scorpiops jendeki *were collected in Yunnan province, Southwest China. They were milked 2 days before RNA isolation as described previously[65]. Total RNA was extracted with TRIZOL Reagent (Invitrogen, Carlsbad, CA, USA), and then mRNA was purified with FastTrack 2.0 mRNA Isolation Kit(Invitrogen). The cDNA library was constructed from 5 μg of mRNA using the Creator SMART cDNA Library Construction Kit (Clontech Laboratories, Palo Alto, CA). cDNA inserts were directionally cloned into the plasmids pDNR-LIB digested by restriction enzymes Sfi IA and Sfi IB. The recombinant plasmids were transformed into electrocompenent *Escherichia coli *DH10B (Invitrogen).

### Sequencing

To obtain an unbiased overview of the venom gland transcriptome, random colonies were selected and cultured in appropriate Luria Broth culture medium containing 30 μg/ml of chloramphenicol. After overnight culture, plasmid DNA was isolated using alkaline lysis method. Purified plasmids were single-pass sequenced on an ABI 3730xl sequencer using the standard M13 forward primer and BigDye terminator v3.1 cycle sequencing kit (Applied Biosystems, Foster City, CA, USA).

### Bioinformatics analysis

The trace files of sequenced clones were subjected to Phred program, the cutoff Phred score was set to 40[66]. After these sequences were strictly trimmed, the got high-quality sequences were processed on the website EGassembler  with the default parameter[17]. Vector and adaptor sequences were removed using the program Cross_Match. After removing the PolyA tail, we discarded those sequences shorter than 100 bp. The resulted sequences were deposited into the dbEST, and then assembled into clusters with the program CAP3.

Each cluster was annotated by being searched against SWISS-PROT  and GenBank NCBI database  with BLAST algorithms. After BLAST search, the unmatched clusters were further identified for open reading frames using the ORFfinder . Considering the extreme diversity of scorpion toxins, those clusters putative to encode venom peptides was reexamined manually to pick out individual different isoforms.

All clusters were checked for the existence of signal peptides using the SignalP 3.0 program . All types of venom peptides and proteins are annotated by searching against Pfam protein families database .

### Alignment and phylogeny analysis

The sequences used for alignment and phylogeny analysis were retrieved from SWISS-PROT databsae . The alignment was performed by Clustal_X 1.83 software followed by manual adjustment[67], and viewed by the software Jalview[68]. Phylogeny analysis was carried out with Neighbor joining method implemented in MEGA3.1[69].

## Authors' contributions

YM carried out cDNA library construction, paticipated in the bioinformatics and phylogeny analysis, and drafted the manuscript. RZ participated in the alignment and phylogeny analysis, and drafted the manuscript. YH participated in the sequencing. SL participated in the sequencing. JL participated in the alignment and phylogeny analysis. YW participated in the design and coordination of the study. ZC participated in the design and coordination of the study, and drafted the manuscript. WL conceived of the study, and participated in its coordination. All authors have read and approved the final manuscript.

## Supplementary Material

Additional file 1 **Atypical venom molecules characterized from the scorpion *Scorpiops jendeki***. The data represents eight novel types of venom peptides encoded by medium-abundant clusters from the scorpion *Scorpiops jendeki*. Click here for file
