# Supplementary material for: Transcriptome analysis of the venom gland of the scorpion Scorpiops jendeki: implication for the evolution of the scorpion venom arsenal
Source: BMC Genomics. 2009 Jul 1;10:290. doi: 10.1186/1471-2164-10-290 (PMC2713264; doi:10.1186/1471-2164-10-290)
Supplement: Additional file 1 — Atypical venom molecules characterized from the scorpion Scorpiops jendeki. The data represents eight novel types of venom peptides encoded by medium-abundant clusters from the scorpion Scorpiops jendeki. [file 1471-2164-10-290-S1.doc]

**Supplementary file**

**Table. Atypical venom molecules characterized from the scorpion *Scorpiops jendeki*.**

| CLUSTERS | ESTs | Sequences |
| --- | --- | --- |
| SJE006C/SJE043C | 14 | >SJE006C  MKVLPVILLALIVLISIPSETLGQIHQDQETDYLPPASKRSAFLRRTARRDDGSPRKR#  >SJE043C  MKVLPVIFLTLIVLITIPSEILAPTHPHCILSFGRKRSALLRRVASRDGGSNPPRKR# |
| SJE077C | 12 | >SJE077C.1  MRGFFLLALILTVQLISCFGARISDEEMAETSYRASEDSAPMHESIRKSIEKRCTIGRCPGFGLLHG#  >SJE077C.2  TNKADKINLALILTVQLISCFGARISDEEMAETSYRASEDSAPMHESIRKSIEKRCTIGRCPGFGLLHG#  >SJE077C.3  MRGFFLLALILTVQLISCFGARISDEEMAETSYRASEDSAPMHESIRKSIDKRCTIGRCPGFGLLHG# |
| SJE055C | 9 | >SJE055C.1  MNYSLTLVLLSVVFCCVLSDEEQLTAGGRYCATRCNRNEVVACTLNHFPEGTNIYVKCASAFDSSVKNLEQSNDFLCNKANPMQFKYIEFCWRHLLARHYPMEDIRKVVYECLDENDC&  >SJE055C.2  MKYSLTLVLLSVVFCCVLSDEEQLTAGSRYCATRCNRNEVVACTLNHFQEGTNIYVKCASAFDSSVKNLEQSNDFLCNKANPMQFKYIEFCWRHLLARHYPMEDIRKVVYECLDENDC& |
| SJE061C | 9 | >SJE061C.1  MKTLPVIFLCPLVLLAAPSGIWCREEMEYQTKNWPNSYNKGKRSMPSINKRRVDILSAPEKDDGI#  >SJE061C.2  MKTLPVIFFFVCSSYWLHHREFGAEKKWSTRPRTGPTLITKGNGLCP&  >SJE061C.3  MKTLPVIFLCLLVLLAVTPGIWCREEMEYQTKNWPNSYNKGKRSMPSINKRRVDMLSAPEKDDGI#  >SJE061C.4  MKTLPVIFLCLLVLLAAPSGIWCREEMEYIPTFYWAWKRSMPSINERRVDILSTSEREEGN#  >SJE061C.5  MKTLPLIFVCLLILLAAPSGIWCREEMEYQTKNWPNSYNKGKRSMPSINKRGVDILSAPEKDDGI#  >SJE061C.6  MKTLPVIFLCLLVLLAAPSGIWCREEMEYQTKNWPNSYNKGKRNMPSINKRRVDILSAPEKDDGI#  >SJE061C.7  MKTLPVIFLCLLVLLAAPSGIWCREEMEYQTKNWPNSYNKGKRSMPSINKRRVDILSAPEKDDGI# |
| SJE033C | 7 | >SJE033C.1  MKPSHCFILVLLVLLPSLLSTASSESRNPPLNGSMFGKRSYTQVEGSEEARAMKCQAYLERCLKLMPAACV#  >SJE033C.2  MKPSHCFILVLLVLLPSLLSTASSESKNPPLNGSMFGKRSYTQVEGSEEARAMKCQAYLERCLKLMPAACV#  >SJE033C.3  MKPSHCFILVLLVLLPSLLSTASSESRNPPLNGSMFGKRSYTQVEGSEEARAMKCQAYLERCLKLTPAACV# |
| SJE025C | 7 | >SJE025C.1  MKMDLCILNFTVIVLISLCANVFAHQLTDDHWEFVCASPNKTVLEIIDCATKLETPYFLRKIKGVQECTRMTQIEIIKSVCVYDSLPQEKVELIDTCVNKYYVESDEELPNPAVGCIERLPAYKSTMDKKQ#  >SJE025C.2  MKMDLCILNFTVIVLISLCANVFAHQLTDDHWEFVCASPNKTVLEIIDCATKLETPYFLKKIKGVQECTRMTQIEIIKSVCVYDSLPQEKVELIDTCVNKYYVESDEELPNPAVGCIERLPAYKSTMDKKQ# |
| SJE038C | 6 | >SJE038C  MKLRVIAFVLIFSIIFTVIQGTNIGKRTSIKRQSNPEQNYELQQQKKCFEFCMGSGSVTFKECQQNCKMPG# |
| SJE044C | 6 | >SJE044C  MKTMFAAVVLAALCAVSMAGYLGAGFGGAGFGGRDAGVTVSFDNQRAAPYGGLGLGYGGLGYAGLGYGGLGLAHGGYGLGLGYGAYGFGRGLGYGVGLGHGLGYGAGLGKIW# |
